# Improving care for pregnant women with suspected influenza: A retrospective study before and after introduction of a rapid molecular assay

**DOI:** 10.1371/journal.pone.0217651

**Published:** 2019-06-14

**Authors:** Olivia Anselem, Camille Baraud, Anne-Sophie L’Honneur, Camille Gobeaux, Flore Rozenberg, François Goffinet

**Affiliations:** 1 Maternité Port-Royal, Groupe hospitalier Cochin Broca Hôtel-Dieu, Assistance Publique Hôpitaux de Paris, Université Paris Descartes, DHU Risques et Grossesse, PRES Sorbonne Paris Cité, Paris, France; 2 Laboratoire de Virologie, Groupe hospitalier Cochin Broca Hôtel-Dieu, Assistance Publique Hôpitaux de Paris, Université Paris Descartes, DHU Risques et Grossesse, PRES Sorbonne Paris Cité, Paris, France; 3 Laboratoire de Diagnostic Biologique Automatisé, Groupe hospitalier Cochin Broca Hôtel-Dieu, Assistance Publique Hôpitaux de Paris, Université Paris Descartes, Paris, France; Stony Brook University Health Sciences Center School of Medicine, UNITED STATES

## Abstract

**Objectives:**

During winter, after excluding obvious sites of infection, the most important diagnoses of isolated fever or influenza-like illness (ILI) to rule out are listeriosis and influenza, because of their severe potential outcomes and the straightforward management available for each. While awaiting laboratory results, the recommended management strategy is usually hospitalization for intravenous antibiotic therapy against potential listeria. This study sought to assess the effect of the use of a rapid test on hospitalization and antibiotic therapy rates.

**Methods:**

The study included all pregnant women who consulted for ILI or isolated fever after clinical and laboratory investigations and had a molecular diagnostic assay for influenza during two time periods, both during influenza epidemics: before introduction of the rapid molecular assay use (period 1) and after this (period 2).

**Results:**

The study included 38 women during period 1 and 124 during period 2. The influenza diagnosis was confirmed for 24 of 38 (63.2%) women during period 1 and 65 of 124 (52.4%) women during period 2 (*P* = 0.24). The hospitalization rate fell significantly from period 1 to period 2, both in the total population (71.0% versus 44.3%, *P* = 0.004) and among women with confirmed influenza (83.3% versus 38.5%, *P*<0.001), as did the antibiotic therapy rate in both groups (respectively, 86.8% versus 56.1%, *P* = 0.001 and 91.7% versus 44.7%, *P*<0.001).

**Conclusion:**

The use of a rapid molecular assay for the diagnosis of influenza improved the management of pregnant women with an isolated fever or ILI by reducing the rates of unnecessary hospitalization and antibiotic therapy.

## Introduction

Fever during pregnancy is a frequent reason for an emergency consultation during the winter. After excluding obvious sites of infection, the most important diagnoses of isolated fever or influenza-like illness (ILI) to rule out are listeriosis and influenza—two infections that can lead to maternal or fetal complications and must be managed differently for pregnant women. The management of listeriosis during pregnancy is based on inpatient intravenous antibiotic therapy, while influenza in pregnancy, in the absence of signs of severity, is treated on an outpatient basis by oral antiviral drugs. The clinical signs of listeriosis are unspecific and sometimes resemble those of an ILI. Because clinical examination cannot distinguish listeriosis from influenza, the differential diagnosis depends on laboratory tests. While awaiting this laboratory confirmation, pregnant women with a fever or ILI are generally hospitalized for antibiotic therapy, because of the severity of the perinatal complications of listeriosis. These include late miscarriage or preterm delivery in more than 50% of cases and can lead to fetal or neonatal death in 25 to 50%.[1] The bacteriological diagnosis of listeriosis is based on the identification of the bacteria from blood cultures, which take 48 to 72 hours to produce a result. Until recently, the molecular diagnosis of influenza required the separate technical steps of nucleic acid extraction and then amplification, most often performed in series only on weekdays during working hours, which delayed results. Automated individual rapid molecular assays of respiratory samples now provide results in less than 2 hours, which enables outpatient antiviral treatment and thus avoids unnecessary hospitalization and antibiotic therapy. Moreover, several studies have shown that the rapid start of antiviral treatment in pregnant women with influenza reduces the risk of maternal and fetal complications from this disease.[2,3]

This study seeks to confirm our hypothesis that the diagnosis of influenza by this rapid assay would improve the management of pregnant women consulting for isolated fever or ILI, by reducing the number of ultimately unnecessary admissions and antibiotic treatments.

## Methods

This retrospective comparative before-and-after single-center study took place at the Port-Royal maternity hospital. The study population comprised the pregnant women with isolated fever or ILI who had a PCR test at the emergency department (ED) to diagnose influenza during the annual influenza epidemic periods from December 2012 through March 2017. These women were identified from the virology laboratory’s computerized register and crossed with the list of women hospitalized during the study periods for an infection to ensure that the women hospitalized for an unexplained fever had indeed been tested for influenza and listeriosis. Fever was defined as a temperature of 38°C or higher. ILI was defined by one or more of the following symptoms: a fever at home, shivering, muscle soreness or pain, headache, cough, or asthenia. All pregnant women presenting at the ED for fever had complete clinical and obstetric examinations and laboratory tests if needed. Women were excluded when the ED visit revealed that the fever was due to another evident infection site, such as a urinary tract infection or chorioamnionitis.

Influenza infection was diagnosed on nasopharyngeal samples (Sigma-Virocult). From December 2012 to March 2014, a standard real-time PCR was performed, targeting the genes for the Influenzavirus A matrix protein and the Influenzavirus B non-structural protein 1 (National Reference Centre, Pasteur Institute, Paris). This qualitative duplex RT-PCR system used the forward primer 5’- CTT CTA ACC GAG GTC GAA ACG TA -3’, the reverse primer 5’- GGT GAC AGG ATT GGT CTT GTC TTT A -3’, and the 6-carboxyfluorescein (FAM)-labeled probe 5’- TCA GGC CCC CTC AAA GCC GAG -3’ to detect Influenzavirus A, and the forward primer 5’- CCT AAC CTC ACT CTT CGA GCG -3’, the reverse primer 5’- CGG TGC TCT TGA CCA AAT TGG -3’, and the VIC-labeled probe 5’- CAA TTC GAG CAG CTG AAA CTG CGG TG -3’ to detect Influenzavirus B. The influenza A and B virus genomes were amplified after nucleic acid extraction with the QIAamp Viral RNA Mini Kit (Qiagen). The result of this test was delivered to the maternity unit staff within around 6 to 24 h on weekdays. Samples were not processed after hours (nights or weekends).

Since January 2015, the Focus Simplexa Flu A/B & RSV Direct assay has been used, in accordance with the manufacturer’s technical instructions. Briefly, this all-in-one reaction test detects and differentiates influenza A, influenza B, and respiratory syncytial virus (RSV) from crude samples in approximately two hours.

Two groups were compared: women tested before the rapid molecular assay became available, that is, between December 2012 and March 2014 (period 1), and those tested after use of the rapid assay began, from January 2015 to March 2017 (period 2). During these two periods, we studied women tested only during influenza epidemics, as the rapid assay was available only during epidemic periods. Dates of influenza epidemic periods were collected from the Institut national de Veille Sanitaire (National Institute of Health Surveillance) (InVS); for period 1 they ran from December 17, 2012, to March 17, 2013, and from January 27, 2014, to March 2, 2014, and for period 2, from January 12, 2015, to April 13, 2015, from January 23, 2016, to May 2, 2016, and from December 14, 2016, to March 30, 2017. The duration of period 1 was thus 18 weeks, and the duration of period 2 was 42 weeks.

Since 2012, the protocol for the management of pregnant women seen at the ED for an isolated fever or ILI during an influenza epidemic period has called for hospitalization and IV antibiotic treatment against potential listeria. Treatment was subsequently adapted according to the results of the blood cultures for listeriosis and nasopharyngeal samples for influenza. When influenza was diagnosed and no signs of severity observed, oseltamivir administration began if it had not previously, the IV antibiotic treatment was stopped, and the woman discharged home. Women vaccinated against influenza were managed just like those who had not been vaccinated. During period 2, the management of pregnant women visiting the ED for an isolated fever or ILI did not change, except that nasopharyngeal samples taken in the ED were tested immediately, 24/7, with results within 2 hours. Unless signs of severity were present, women with positive tests for influenza received antiviral treatment on an outpatient basis, and those whose tests were negative were admitted, had blood cultures run, and received IV antibiotic therapy against potential listeriosis.

The principal endpoint was the hospitalization rate for the entire study population. The secondary outcome measures were the hospitalization rate among women with confirmed influenza, the length of stay, and the rates and durations of antibiotic and of antiviral treatment for the entire study population and for those with confirmed influenza.

We collected time until results, defined by the interval between the laboratory’s receipt of the sample and the time the result was available to the clinician. Threatened preterm delivery was defined by uterine contractions and cervical modifications. The fetal heart rate (FHR) abnormalities were tachycardia, defined by a baseline FHR ≥ 160 bpm, the presence of decelerations, the absence of acceleration, and micro-oscillation. Obstetric and neonatal outcomes were also collected, particularly maternal infectious complications after discharge, gestational age at birth, preterm birth rate, intrauterine fetal death or perinatal death, and birth weight.

The statistical analyses were performed with Chi^2^ tests for the qualitative variables and Student’s t test for normally distributed quantitative variables with Stata software (version 11.0).

This study was approved by the National Data Protection Authority (Commission Nationale de l’Informatique et des Libertés, CNIL n°1755849). Under French regulations, this study is exempt from IRB review because it is an observational study using anonymized data from medical records. Women are informed that their records can be used for the evaluation of medical practices and are provided the option to opt out these studies.

## Results

Overall, 162 women consulted for isolated fever after exclusion of other sites infection or ILI and had a diagnostic test for influenza during the study period: 38 during period 1 and 124 during period 2.

The groups did not differ significantly for maternal age, number of pregnancies, parity, body mass index, smoking status, asthma, or type 1 diabetes (Table 1). The rate of vaccination against influenza was lower during period 1: 2.6% compared with 19.3% during period 2 (*P* = 0.01).

**Table 1:** General characteristics and clinical signs of women who consulted for an isolated fever or ILI at emergency department visit.

	Period 1 n = 38	Period 2 n = 124	*P* value
**Mean age, years (mean, standard deviation)**	31.6 (+/-5.4)	32.2(+/-5.2)	0.74
**Number of pregnancies (mean, standard deviation)**	2.5 (+/-1.7)	2.5(+/-1.6)	0.98
**Parity (mean, standard deviation)**	0.9 (+/-1.1)	0.9 (+/-1.1)	0.92
**BMI, kg/m^2^ (mean, standard deviation)**	24.4 (+/-4.7)	23.9 (+/-5.7)	0.64
**Diabetes (type 1) (n; %)**	1 (2.6)	13 (10.5)	0.27
**Smoker (n; %)**	2 (5.3)	10 (8.1)	0.56
**Asthma (n; %)**	2 (5.3)	10 (8.1)	0.56
**Influenza vaccination (n; %)**	1 (2.6)	24 (19.3)	0.01
**Gestational age (mean, standard deviation; years)**	30.5 (+/-6.3)	29.0 (+/-7.2)	0.25
**Fever > 38°C (n; %)**	32 (84.1)	103 (83.1)	0.87
**ENT signs (n; %)**	29 (76.3)	92 (74.2)	0.79
ILI* (n; %)	31 (81.6)	67 (54.0)	0.01
**GI signs (n; %)**	4 (10.5)	17 (13.7)	0.61
**TPD (n; %)**	4 (10.5)	8 (6.4)	0.40
**Abnormal FHR: tachycardia(n; %)**	7 (18.4)	19 (15.3)	0.65
**other abnormal FHR (n; %)**	2 (5.3)	3 (2.4)	0.37

ENT: ear, nose, and throat. FHR: fetal heart rate. ILI: influenza-like illness. GI: gastrointestinal. TPD: threatened preterm delivery.

* one or more signs including: fever at home, shivering, muscle soreness or pain, headaches, coughing, asthenia.

There was no significant difference in gestational age at the ED visit or in the existence of fever, ENT signs, or GI signs (Table 1). The rates of threatened preterm delivery and FHR abnormalities were similar during the 2 periods.

No influenza diagnostic tests were performed after hours during period 1, compared with 56.4% during period 2 (*P*<0.001). The mean time to result availability was significantly longer during period 1 than period 2: 20.3 (± 15) hours versus 4.9 (±5) hours (*P*<0.001).

Listeriosis was diagnosed in one woman in period 1 and none in period 2. Eighty-nine women had confirmed influenza: 24 of 38 women (63.2%) during period 1 and 65 of 124 (52.4%) during period 2 (*P* = 0.24).

In the overall population, the hospitalization rate was higher during period 1 than period 2: 71.0% versus 44.3% (*P* = 0.004) (Table 2). The antibiotic treatment rate was also higher during period 1: 86.8% versus 56.1% (*P* = 0.001).

**Table 2:** Results of influenza test and obstetric and neonatal outcomes.

	Period 1 n = 38	Period 2 n = 124	*P* value
After-hours* (n; %)	0	70 (56.4)	<0.001
**Time to result, hours (mean, standard deviation)**	20.3 (+/-15)	4.9 (+/-5)	<0.001
**PCR influenza positive (n; %)**	24 (63.2)	65 (52.4)	0.24
** Influenza A**	16	48	
** Influenza B**	8	15	0.69
** RSV**	0	2	
**Hospitalization (n; %)**	27 (71.0)	55 (44.3)	0.004
** duration (days; min-max)**	4.2 (+/-3.8)	3.8 (+/-2.6)	0.28
**Antibiotic treatment (n; %)**	33 (86.8)	69 (56.1)	0.001
** duration (days)**	4.9 (+/-3.2)	4.8 (+/-4.7)	0.80
**Oseltamivir (n; %)**	26 (68.4)	72 (58.1)	0.25
** duration (days)**	5.1 (+/-1.9)	4.7 (+/-1.0)	0.31
**Gestational age at birth (weeks of gestation) (mean; standard deviation)**	39.3 (+/-2.6)	39.0 (+/-3.2)	0.25
**Preterm birth (n; %)**	3 (7.9)	7(5.6)	0.34
**Weight (grams) (mean; standard deviation)**	3221 (+/-611)	3182(+/-734)	0.52

*diagnostic test performed after hours (18h-8h, weekends, and holidays)

Among the women with confirmed influenza, the hospitalization rate also fell from during period 1 to period 2: 83.3% versus 38.5% (*P*<0.001) (Table 3), as did the antibiotic treatment rate: respectively 91.7% versus 44.7% (*P*<0.001). There was no significant difference for length of stay. The mean duration of antibiotic therapy was longer during period 1 than period 2.

**Table 3:** Management of women with confirmed influenza.

	Period 1 n = 24	Period 2 n = 65	*P* value
**Hospitalization (n; %)**	20 (83.3)	25 (38.5)	<0.001
** duration (days)**	3.2 (+/-2.1)	3.3 (+/-2.6)	0.96
**Antibiotic treatment (n; %)**	22 (91.7)	29 (44.7)	<0.001
** duration (days)**	3.8 (+/-2.4)	1.9 (+/-1.6)	0.001
**Oseltamivir (n; %)**	20 (83.3)	62 (95.4)	0.06
** duration (days)**	5.7 (+/-1.7)	5.0 (+/-0.4)	0.008

No maternal or fetal complication due to influenza was observed after discharge in both groups. Gestational age at birth, preterm birth rate, and birth weight were similar between the two periods.

## Discussion

The use of a rapid molecular assay for influenza was associated with a reduction in the hospitalization rate for pregnant women consulting at the ED for isolated fever or ILI. This test thus makes it possible to avoid unnecessary hospitalization and antibiotic treatment in pregnant women with confirmed influenza, who account for more than half of this population. Moreover, determining the correct diagnosis—listeriosis or influenza—allows management appropriate for the actual maternal and fetal risks.

This strategy of use of the rapid diagnostic test was also assessed in the ED in a population of both children and adults with risk factors for severe influenza in 2016: it enabled 10.7% of hospitalizations to be avoided, and 46.4% of antibiotic prescriptions.[4] To our knowledge, our study is the first to examine the use of a rapid molecular test for influenza available on an emergency basis in a population of pregnant women.

Review of the records, one by one, enabled the collection of specific data. During the study period, our protocol for the management of pregnant women visiting the ED for an isolated fever or ILI did not change, except for the 24/7 use of this rapid molecular assay for influenza. The characteristics of the women during the ED visit were comparable between the two periods, in particular for the rate of threatened preterm delivery and for fetal heart rate abnormalities. This lack of difference for these complications means that these potential additional reasons for hospitalization are unlikely to have introduced bias into our results. Moreover, we note that few women presented any signs of severity during their ED visit; this suggests that most of them could be managed for influenza on an outpatient basis.

However our study has some limitations. First, this is a retrospective study with limited number of women. Furthermore, the number of women included in the study during period 2 was higher than during period 1, essentially because of the difference in the durations of the two exposure periods: 42 and 18 weeks respectively. It is probable that during period 2, women underwent a diagnostic test for influenza more routinely both because the ED staff had developed the habit of using this test, and because the availability of a rapid result might have served as an incentive for using the test more systematically. This also explains the higher proportion of ILI among the women tested during period 1 compared with period 2. It was not possible to find all of the women who came to the ED for these symptoms during the study periods and who were not tested for influenza. Therefore we cannot know if the proportion of women tested for influenza among women consulting in the ED for fever or ILI was the same in both periods. Nonetheless we were able to verify that all of the women hospitalized for an isolated fever or ILI did indeed have a diagnostic test for influenza, based on the list of women hospitalized for fever, also obtained from a computerized register. Another limitation of our study could be differences in infectivity and severity in circulating strains of influenza infection, which could make epidemic periods not comparable. We also noted that the rate of vaccination against influenza was different between the two periods. Nonetheless, women were managed identically regardless their history of vaccination against influenza.

The data in the literature suggest that beginning antiviral treatment within 48 hours of the onset of influenza makes it possible to reduce its complication rate.[2] The time to results was, as expected, longer during period 1 than period 2, due to the use of the rapid assay. Nonetheless, the moment that treatment began was not available. However, we can suppose that the use of this test also makes it possible to reduce the time until antiviral treatment starts. The use of a rapid molecular test for influenza has several other major advantages, including the avoidance of futile probabilistic antibiotic therapy. The antibiotic treatment against listeriosis is generally based on high doses of ampicillin. Although not associated with a risk of fetal malformation,[5] antibiotic use can have consequences on the individual scale on the microbiota[6,7] and can modify the bacterial ecosystem.[8,9] The prescription of antibiotics during pregnancy must therefore be sustainable.

Finally, we did not conduct a formal medical economic evaluation of the rapid test. The higher cost of the rapid test is probably largely offset by the reduction in the overall costs of management. In their study published in 2017 on the management of patients with influenza symptoms during the 2013–2014 epidemic season, Houssan et al. showed that the use of multiplex PCR, compared with the RIDT technique, reduced the cost of management in one large hospital in one influenza epidemic by $208,000.[10]

## Conclusion

The use of a rapid molecular assay for the diagnosis of influenza during epidemics improved the management of pregnant women with an isolated fever or ILI by reducing the rates of unnecessary hospitalization and antibiotic therapy. These results confirmed our decision to adopt this rapid test in everyday practice.
